# Genetic variants associated with fasting glucose and insulin concentrations in an ethnically diverse population: results from the Population Architecture using Genomics and Epidemiology (PAGE) study

**DOI:** 10.1186/1471-2350-14-98

**Published:** 2013-09-25

**Authors:** Megan D Fesinmeyer, James B Meigs, Kari E North, Fredrick R Schumacher, Petra Bůžková, Nora Franceschini, Jeffrey Haessler, Robert Goodloe, Kylee L Spencer, Venkata Saroja Voruganti, Barbara V Howard, Rebecca Jackson, Laurence N Kolonel, Simin Liu, JoAnn E Manson, Kristine R Monroe, Kenneth Mukamal, Holli H Dilks, Sarah A Pendergrass, Andrew Nato, Peggy Wan, Lynne R Wilkens, Loic Le Marchand, José Luis Ambite, Steven Buyske, Jose C Florez, Dana C Crawford, Lucia A Hindorff, Christopher A Haiman, Ulrike Peters, James S Pankow

**Affiliations:** 1 Division of Public Health Sciences, Fred Hutchinson Cancer Research Center, Seattle WA, USA; 2 General Medicine Division, Department of Medicine, Harvard Medical School, Boston MA, USA; 3 Carolina Center for Genome Sciences, School of Public Health, University of North Carolina, Chapel Hill, NC, USA; 4 Department of Epidemiology, School of Public Health, University of North Carolina at Chapel Hill, Chapel Hill, NC, USA; 5 Department of Preventive Medicine, Keck School of Medicine / Norris Comprehensive Cancer Center, University of Southern California, Los Angeles, CA, USA; 6 Department of Biostatistics, University of Washington, Seattle, WA, USA; 7 Center for Human Genetics Research, Vanderbilt University Medical Center, Nashville, TN, USA; 8 Department of Genetics, Texas Biomedical Research Institute, San Antonio, TX, USA; 9 MedStar Research Institute, Georgetown University, Hyattsville, MD, USA; 10 Department of Internal Medicine, Ohio State Medical Center, Columbus, OH, USA; 11 Epidemiology Program, University of Hawaii Cancer Center, Honolulu, HI, USA; 12 Department of Epidemiology, University of California, Los Angeles, CA, USA; 13 Brigham and Women’s Hospital, Harvard Medical School, Boston, MA, USA; 14 Beth Israel Deaconess Medical Center, Harvard Medical School, Boston, MA, USA; 15 Department of Biochemistry and Molecular Biology, Pennsylvania State University, University Park, PA, USA; 16 Department of Genetics, Rutgers University, Piscataway, NJ, USA; 17 Information Sciences Institute, University of Southern California, Marina del Rey, CA, USA; 18 Department of Statistics & Biostatistics, Rutgers University, Piscataway, NJ, USA; 19 Office of Population Genomics, National Human Genome Research Institute, National Institutes of Health, Bethesda, MD, USA; 20 Division of Epidemiology and Community Health, University of Minnesota School of Public Health, Minneapolis MN, USA

## Abstract

**Background:**

Multiple genome-wide association studies (GWAS) within European populations have implicated common genetic variants associated with insulin and glucose concentrations. In contrast, few studies have been conducted within minority groups, which carry the highest burden of impaired glucose homeostasis and type 2 diabetes in the U.S.

**Methods:**

As part of the 'Population Architecture using Genomics and Epidemiology (PAGE) Consortium, we investigated the association of up to 10 GWAS-identified single nucleotide polymorphisms (SNPs) in 8 genetic regions with glucose or insulin concentrations in up to 36,579 non-diabetic subjects including 23,323 European Americans (EA) and 7,526 African Americans (AA), 3,140 Hispanics, 1,779 American Indians (AI), and 811 Asians. We estimated the association between each SNP and fasting glucose or log-transformed fasting insulin, followed by meta-analysis to combine results across PAGE sites.

**Results:**

Overall, our results show that 9/9 GWAS SNPs are associated with glucose in EA (p = 0.04 to 9 × 10^-15^), versus 3/9 in AA (p= 0.03 to 6 × 10^-5^), 3/4 SNPs in Hispanics, 2/4 SNPs in AI, and 1/2 SNPs in Asians. For insulin we observed a significant association with rs780094/*GCKR* in EA, Hispanics and AI only.

**Conclusions:**

Generalization of results across multiple racial/ethnic groups helps confirm the relevance of some of these loci for glucose and insulin metabolism. Lack of association in non-EA groups may be due to insufficient power, or to unique patterns of linkage disequilibrium.

## Background

Impaired glucose homeostasis is associated with increased risk of cardiovascular disease and type 2 diabetes [1,2]. While plasma glucose and insulin concentrations normally fluctuate in response to dietary intake and physical activity levels, several genome-wide association studies (GWAS) have demonstrated that common genetic variants contribute to glucose homeostasis. Most studies of this complex trait have focused on primarily ancestrally European populations, despite the high prevalence of insulin resistance and diabetes in many minority groups.

Investigation of the clinical and public health implications of these genetic discoveries requires not only confirmation in EA populations, but importantly generalization of these associations to other groups such as African Americans, Hispanics, American Indians, and Asians; groups that were not adequately represented in most GWAS. A prior study found that a genetic risk score composed of 16 SNPs previously associated with fasting glucose in GWAS in European populations was associated with fasting glucose in non-Hispanic blacks and Mexican Americans, indicating that genetic factors associated with fasting glucose may be shared across some racial/ethnic groups [3]. The purpose of this study is to examine 36,579 participants from diverse racial and ethnic backgrounds as part of the NHGRI-supported 'Population Architecture using Genomics and Epidemiology (PAGE)’ Consortium to investigate associations between SNPs previously-identified in genome-wide scans for loci associated with glucose and insulin concentrations.

## Methods

### Study populations

PAGE involves several studies, described briefly below and in greater detail on the PAGE website (https://www.pagestudy.org). All studies were approved by Institutional Review Boards at their respective sites, and all participants provided informed consent.

Causal Variants across the Life Course (CALiCo) is a consortium of six demographically diverse population based studies and a central laboratory, and includes approximately 58,000 men and women ranging in age from adolescence to older adulthood. Three CALiCo studies participated in the present analysis: Atherosclerosis Risk in Communities Study (ARIC) (N = 13,383) [4], Cardiovascular Health Study (CHS) (N = 4,509) [5], and Strong Heart Cohort Study (SHCS) (N = 1,714) [6]. In addition to the studies involved in the CALiCo consortium, PAGE includes three other large studies. The Multiethnic Cohort (MEC) is a population-based prospective cohort study of over 215,000 men and women in Hawaii and California aged 45–75 at baseline (1993–1996) and primarily of five ancestries [7]. Participants eligible for the present study were controls in nested case–control studies of breast, colorectal, or prostate cancer or for biomarker studies, and who had glucose and/or insulin measurements (N=942). This analysis also included data from the Epidemiologic Architecture for Genes Linked to Environment (EAGLE) study. EAGLE accesses the genetic component of three National Health and Nutrition Examination Surveys (NHANES): NHANES III (phase 2 collected between 1991 and 1994), NHANES 1999–2000, and NHANES 2001-2002 [8-10]. Overall, 7,719 NHANES participants aged 18 and older were included in these analyses. Finally, the Women’s Health Initiative (WHI) is a multifaceted clinical trial and cohort study investigating post-menopausal women’s health in the U.S [11]. Out of the 161,808 women enrolled in WHI, 8,312 were selected and included in the present study. Except for the Women’s Health Initiative, all studies recruited men and women. All studies collected self-identified racial/ethnic group via questionnaire. In the current analysis, we included "East Asians" defined as MEC participants who identified themselves as of sole or mixed Japanese descent, and WHI participants of Japanese, Chinese, Filipino, Vietnamese, and/or Korean ancestry. Fasting glucose and insulin concentrations were measured using standard assays, at laboratories specific to each PAGE site.

At all PAGE sites, we excluded underweight (BMI<18.5 kg/m^2^) and extremely overweight (BMI>60 kg/m^2^) individuals with the assumption that these extremes could be attributable to data coding errors, an underlying illness or possibly to a familial syndrome and hence, a rare mutation. We excluded individuals self-reporting that they have ever been diagnosed with diabetes, or who report taking diabetes medications. In addition, to mirror typical exclusion criteria of other studies of glucose homeostasis, we also excluded individuals with fasting glucose concentrations consistent with diabetes (i.e., ≥126 mg/dl or ≥7.0 mmol/L ), regardless of self-reported diabetes status.

After applying the above exclusion criteria, a total of 36,579 participants were selected from the PAGE consortium for analysis.

### SNP selection and genotyping

Ten SNPs in 8 genetic regions were selected for genotyping based on prior GWAS findings of positive association with glucose or insulin concentrations, and exceeding a genome-wide significance of p <5 × 10^-8^ in studies published through 2010 [12-14]. Nine SNPs were previously associated with glucose, and 2 were associated with insulin, with 1 of these SNPs associated with both quantitative traits (rs780094/*GCKR*). In the glucose analysis, we included an additional GWAS finding for type 2 diabetes (rs7903146/*TCF7L2*) that had been subsequently associated with fasting glucose concentrations [15]. Each PAGE site prioritized which SNPs to genotype based on investigator interests, genotyping platforms, and resources, resulting in heterogeneity of available glucose or insulin SNPs across racial/ethnic groups. Ten SNPs were genotyped in European Americans and African Americans, 4 were genotyped in Hispanics and in American Indians, and 2 were genotyped in East Asians.

DNA extraction and genotyping methods followed standard protocols. Each PAGE site employed different genotyping platforms, with similar quality control criteria. CALiCo sites used TaqMan, the Illumina 370CNV BeadChip, the Affymetrix Genome-Wide Human SNP Array 6.0, and the Illumina HumanCVD BeadChip. A portion of CHS genotype data was obtained from a previous GWAS. EAGLE used Sequenom’s iPLEX® Gold coupled with MassARRAY MALDI-TOF MS detection and Illumina’s BeadXpress with a custom GoldenGate genotyping assay. MEC used Applied Biosystems OpenArray and TaqMan. WHI used Illumina BeadXpress with the Veracode GoldenGate genotyping assay. All sites used internal and blinded external controls, and excluded genotypes deviating from Hardy-Weinberg expectations (p-value < 0.001) or with low concordance (typically, <95% - 99%). In addition to site-specific quality control, all PAGE study sites genotyped 360 DNA samples from the International HapMap Project and submitted these data to the PAGE Coordinating Center for concordance checks [16]. Additional details on data collection, specimen processing, and genotyping are found in the Additional file 1: Supplementary Methods.

### Statistical analysis

In order to maximize comparability with prior studies of glucose homeostasis, we converted insulin and glucose concentrations into units commonly reported in the literature. Thus, we investigated continuous fasting glucose (mmol/L) and natural log transformed fasting insulin (pmol/L). The association between each SNP and its related quantitative trait was estimated using linear regression with robust standard errors (SEs) [17]. SNP genotype was coded assuming an additive genetic model (i.e., 0, 1, or 2 copies of the coded allele). For ease in interpreting the results, we coded the allele that was associated with an increased insulin or glucose concentration in the prior GWAS. All analyses were stratified by self-identified racial/ethnic group, and adjusted for covariates known to be associated with insulin and/or glucose concentrations: smoking (current vs. former/never; smoking increases insulin resistance) [18], continuous BMI (obesity is associated with insulin resistance) [19], sex (insulin metabolism differs by sex) [20], and continuous age (insulin metabolism varies by age) [21]. Analyses were performed for each of the 6 participating PAGE studies separately and study-specific results (effect sizes and robust SEs) were combined with fixed-effects meta-analysis using R.

Based on our hypothesis that GWAS-identified glucose and insulin SNPs are associated with glucose and/or insulin concentrations across all race/ethnicities, we did not adjust for multiple testing. We labeled meta-analysis results as "replicating" (for EA) or "generalizing" (for other racial/ethnic groups) if the beta was in the same direction as the original GWAS, and was statistically significant (i.e., p < 0.05). All aggregate results will be available via dbGaP (http://www.ncbi.nlm.nih.gov/gap) at a future date.

Approximately 13% of the overall WHI study cohort was selected to contribute to PAGE. This selection was non-random, and was enriched for subjects with certain incident health conditions (e.g., cardiovascular disease and stroke), non-European American race/ethnicity, and BMI>40. Therefore, analyses of WHI data incorporated inverse probability weighting to account for this sampling strategy.

We only reported results if the meta-analysis sample size was > 400. For each racial/ethnic group, we estimated the statistical power to detect the GWAS-reported effect sizes for each SNP using Quanto (http://hydra.usc.edu/gxe/), assuming the same effect size as reported in the prior GWAS, an additive genetic model and a two-sided test of association at p = 0.05. Power calculations were based on allele frequencies specific to each racial/ethnic group. We evaluated I^2^ as a measure of heterogeneity [22], to describe the presence or absence of excess variation across the PAGE study sites.

## Results

The distribution of insulin and glucose measurements and demographic characteristics for participants in each PAGE site by racial/ethnic group are detailed in Table 1. In general, subjects were middle-aged to older adults (mean age ranging from 41 to 73 years across studies), with average BMI ranging from normal to obese (24 – 32 kg/m^2^). Coded allele frequencies, stratified by racial/ethnic group, are presented in Table 2. Analyses involved a total of 36,579 subjects, including 23,323 European Americans, 7,526 African-Americans, 3,140 Hispanics, 1,779 American Indians, and 811 East Asians.

**Table 1:** Characteristics of PAGE participants, by race/ethnicity and PAGE site

**European Americans**							**African Americans**					
**Site**	**N**	**Female (%)**	**Age**	**BMI**	**Fasting glucose**	**Fasting insulin**	**N**	**Female (%)**	**Age**	**BMI**	**Fasting glucose**	**Fasting insulin,**
			**yrs**	**kg/m**^**2**^	**mmol/L**	**pmol/L**			**yrs**	**kg/m**^**2**^	**mmol/L**	**pmol/L**
ARIC	10221	53.4	54.2 (5.7)	26.7 (4.6)	5.5 (0.5)	60.7 (44.7)	3162	61.4	53.2 (5.8)	29.1 (6.0)	5.5 (0.6)	79.7 (58.3)
CHS	3902	58.0	72.6 (5.6)	26.1 (4.3)	5.5 (0.5)	82.4 (42.3)	607	63.1	73.0 (5.8)	27.9 (5.4)	5.5 (0.6)	77.9 (47.4)
EAGLE	3713	54.4	51.1 (19.6)	27.3 (5.7)	5.5 (1.1)	64.3 (42.5)	1791	55.5	41.5 (16.2)	28.6 (6.6)	5.3 (1.3)	75.1 (59.4)
MEC	238	44.2	67.8 (8.1)	25.2 (4.1)	4.7 (0.6)	43.5 (39.4)	157	22.3	67.8 (7.1)	27.3 (4.2)	4.7 (0.9)	43.0 (30.9)
WHI	5249	100.0	67.0 (6.9)	28.0 (6.3)	5.2 (0.5)	52.9 (41.2)	1809	100.0	62.0 (7.1)	31.6 (7.2)	5.1 (0.6)	72.9 (49.0)
**Total**	**23323**						**7526**					
**Hispanics**							**American Indians**					
**Site**	**N**	**Female (%)**	**Age**	**BMI**	**Fasting glucose**	**Fasting insulin**	**N**	**Female (%)**	**Age**	**BMI**	**Fasting glucose**	**Fasting insulin**
			**yrs**	**kg/m**^**2**^	**mmol/L**	**pmol/L**			**yrs**	**kg/m**^**2**^	**mmol/L**	**pmol/L**
EAGLE	2215	50.7	41.6 (17.1)	28.1 (5.4)	5.5 (1.3)	78.1 (55.5)						
MEC	133	33.8	67.5 (6.9)	26.1 (3.3)	4.7 (0.6)	34.9 (31.5)						
SHCS							1714	56.5	55.8 (8.3)	29.4 (6.0)	5.6 (0.6)	87.5 (70.4)
WHI	792	100.0	60.0 (6.7)	28.2 (5.4)	5.1 (0.5)	63.1 (43.5)	65	100.0	62.0 (7.8)	28.8 (5.4)	5.1 (0.6)	64.2 (51.0)
**Total**	**3140**						**1779**					
**East Asians**												
**Site**	**N**	**Female (%)**	**Age**	**BMI**	**Fasting glucose**	**Fasting insulin**						
			**yrs**	**kg/m**^**2**^	**mmol/L**	**pmol/L**						
MEC	414	51.6	68.7 (7.9)	24.3 (3.4)	4.8 (0.6)	41.4 (23.9)						
WHI	397	100.0	65.0 (7.4)	25.1 (4.5)	5.3 (0.5)	44.4 (25.8)						
**Total**	**811**											

Means and standard deviations are shown for continuous characteristics. ARIC: Atherosclerosis Risk in Communities Study; CHS: Cardiovascular Health Study; EAGLE: Epidemiologic Architecture of Genes Linked to Environment; MEC: Multiethnic Cohort; WHI: Women’s Health Initiative; SHCS: Strong Heart Cohort Study; BMI: body mass index; Note: minimum BMI was 18.5 for all sites and ancestry groups.

**Table 2:** Coded allele frequencies for glucose and insulin SNPs, stratified by racial/ethnic group

**SNP**	**SNP location**	**Gene**	**GWAS phenotype**	**CA**	**NCA**	**European Americans**	**African Americans**	**Hispanics**	**American Indians**	**East Asians**
rs11708067	intron	*ADCY5*	glucose	a	g	0.78	0.84	NG	NG	NG
rs560887	intron	*G6PC2*	glucose	c	t	0.71	0.93	0.86	0.92	NG
rs4607517	5’ region	*GCK*	glucose	a	g	0.18	0.07	NG	0.25	NG
rs780094	intron	*GCKR*	glucose and insulin	c	t	0.59	0.81	0.67	0.28	0.45
rs7944584	intron	*MADD*	glucose	a	t	0.73	0.95	NG	NG	NG
rs10830963	intron	*MTNR1B*	glucose	g	c	0.28	0.07	0.23	NG	NG
rs11558471	3’ UTR	*SLC30A8*	glucose	a	g	0.68	0.91	NG	NG	NG
rs4506565	intron	*TCF7L2*	glucose	t	a	0.32	0.45	NG	NG	NG
rs7903146	intron	*TCF7L2*	glucose	t	c	0.29	0.28	0.24	0.12	0.05
rs35767	5’ region	*IGF1*	insulin	g	a	0.84	0.55	NG	NG	NG

CA: coded allele; NCA: non-coded allele; NG: not genotyped; UTR: untranslated region.

### Glucose SNPs

Table 3 lists meta-analysis results for EA and AA, for 9 SNPs previously associated with glucose in GWAS. Within European Americans all coded alleles were significantly associated with increased glucose concentrations, and thus replicated findings of prior GWAS. In African Americans, 7 of 9 SNPs demonstrated associations in the same direction as the original GWAS report, and 3 were statistically significant (rs10830963/*MTNR1B*, p = 3.7 × 10^-4^; rs4506565/*TCF7L2*, p = 0.03; and rs7903146/*TCF7L2*, p = 5.9 × 10^-5^). In Hispanics, 3 out of 4 genotyped SNPs were associated with significantly increased glucose concentrations (rs560887/*G6PC2*, p = 5.5 × 10^-5^; rs780094/*GCKR*, p = 2.7 × 10^-5^; and rs10830963/*MTNR1B*, p = 3.3 × 10^-5^). Two out of four SNPs were also associated with increased glucose concentrations in American Indians (rs4607517/*GCKR*, p = 0.03; and rs780094/*GCKR*, p = 0.04). Only 2 SNPs were genotyped in East Asians, and 1 was associated with increased glucose concentrations (rs780094/*GCKR*, p = 0.03).

**Table 3:** Meta-analysis of selected candidate SNPs and fasting glucose and natural log insulin, by racial/ethnic group

		**Coded**	**European Americans**				**African Americans**				**Hispanics**			
**Phenotype**	**SNP**	**Allele**	Effect Size (95% CI)*	**P-value**	**N**	**Power**	Effect Size (95% CI)*	**P-value**	**N**	**Power**	Effect Size (95% CI)*	**P-value**	**N**	**Power**
glucose	rs11708067	A	0.068 (0.013 - 0.123)	**2.0E-02**	9323	0.86	0.151 (-0.064 - 0.366)	1.7E-01	3080	0.34				
(mmol/L)	rs560887	C	0.072 (0.054 - 0.090)	**9.0E-15**	14946	0.99	0.041 (-0.025 - 0.106)	2.3E-01	4857	0.70	0.100 (0.051 - 0.149)	**5.5E-05**	1666	0.64
	rs4607517	A	0.061 (0.030 - 0.093)	**1.6E-04**	2916	0.95	0.025 (-0.111 - 0.160)	7.2E-01	456	0.13				
	rs780094	C	0.050 (0.036 - 0.064)	**1.2E-12**	21608	0.99	0.026 (-0.014 - 0.065)	2.0E-01	5699	0.51	0.063 (0.029 - 0.098)	**2.7E-05**	1992	0.38
	rs7944584	A	0.055 (0.002 - 0.108)	**4.0E-02**	9191	0.72	-0.177 (-0.544 - 0.191)	3.5E-01	3037	0.09				
	rs10830963	G	0.063 (0.043 - 0.083)	**9.5E-10**	16883	0.99	0.096 (0.043 - 0.149)	**3.7E-04**	6234	0.92	0.076 (0.040 - 0.112)	**3.3E-05**	2223	0.97
	rs11558471	A	0.080 (0.033 - 0.126)	**7.8E-04**	10741	0.96	-0.138 (-0.386 - 0.111)	2.8E-01	3447	0.19				
	rs4506565	T	0.107 (0.059 - 0.154)	**9.8E-06**	10644	0.88	0.160 (0.013 - 0.306)	**3.0E-02**	3425	0.35				
	rs7903146	T	0.052 (0.014 - 0.052)**	**7.1E-03**	21710	0.99	0.050 (0.022 - 0.078)	**5.9E-05**	7469	0.54	0.010 (-0.026 - 0.046)	5.7E-01	2611	0.28
(ln)insulin	rs780094	C	0.034 (0.024 - 0.044)	**1.3E-10**	20945	0.14	0.022 (-0.006 - 0.050)	1.2E-01	5648	0.06	0.039 (0.008 - 0.070)	**1.3E-02**	1975	0.06
(pmol/L)	rs35767	G	0.018 (-0.005 - 0.040)	1.3E-01	10741	0.05	0.022 (-0.011 - 0.057)	1.8E-01	3446	0.05				
		**Coded**	**American Indians**				**East Asians**				**Original GWAS Report**			
**Phenotype**	**SNP**	**Allele**	Effect Size (95% CI)*	**P-value**	**N**	**Power**	Effect Size (95% CI)*	**P-value**	**N**	**Power**	**Reference**	**Effect Size (95% CI)**		
glucose	rs11708067	A									Dupuis et al. †	0.027 (0.021 – 0.033)		
(mmol/L)	rs560887	C	0.128 (-0.047 - 0.303)***	1.5E-01	1298	0.28					Bouatia-Naji et al.	0.060 (0.050 – 0.080)		
	rs4607517	A	0.053 (0.006 - 0.100)	**2.8E-02**	1282	0.63					Prokopenko et al.	0.062 (0.048 - 0.076)		
	rs780094	C	0.048 (0.003 - 0.093)	**3.6E-02**	1739	0.25	0.063 (0.006 - 0.121)	**3.1E-02**	736	0.17	Dupuis et al. †	0.029 (0.023 - 0.035)		
	rs7944584	A									Dupuis et al. †	0.021 (0.015 - 0.027)		
	rs10830963	G									Prokopenko et al.	0.072 (0.062 - 0.082)		
	rs11558471	A									Dupuis et al. † ‡	0.027 (0.019 - 0.035)		
	rs4506565	T									Dupuis et al. † §	0.023 (0.015 - 0.031)		
	rs7903146	T	-0.037 (-0.096 - 0.023)	2.3E-01	1738	0.11	-0.033 (-0.181 - 0.114)	6.6E-01	758	0.06	Dupuis et al. †	0.023 (0.015 - 0.031)		
(ln)insulin	rs780094	C	0.080 (0.035 - 0.125)	**4.8E-04**	1697	0.05	0.026 (-0.033 - 0.085)	3.9E-01	689	0.05	Dupuis et al. †	0.032 (0.024 - 0.040)		
(pmol/L)	rs35767	G									Dupuis et al. †	0.010 (-0.002 - 0.022)		

CI: confidence interval; *Effect size is calculated for coded allele relative to non-coded allele; † effect size from replication cohort, as effect size was not reported for discovery cohort; ‡ effect size reported for rs13266634, a SNP in LD with rs11558471 (r2 = 0.96); § effect size reported for rs7903146, a SNP in LD with rs4506565 (r2 = 0.92);**heterogeneity p-value = 0.001, χ2 = 18.79 in a fixed effects model, so results from a random effects model are shown; ***heterogeneity p-value = 0.03, χ2 = 7.31 in a fixed effects model, so results from a random effects model are shown.

### Insulin SNPs

Table 3 lists meta-analysis results for EA and AA, for 2 SNPs (rs780094/*GCKR* and rs35767/*IGF1*) associated insulin in prior GWAS. The association between rs780094/*GCKR* and (ln) insulin replicated in EA (p-value = 1.3 × 10^-10^), but did not generalize to AA (p = 0.12). This association was also significant in Hispanics (p = 0.01) and American Indians (p = 4.8 × 10^-4^), but not East Asians (p = 0.39). The association between rs35767/*IGF1* and insulin was not significant in EA or AA.

### Evidence for heterogeneity

Overall, we observed little evidence of heterogeneity across studies. In EA, rs7903146/*TCF7L2* had a statistically significant p-value for heterogeneity in the association with glucose (χ^2^ = 18.79, p = 0.001), For this association, the site-specific betas for WHI, CHS, EAGLE, ARIC, and MEC were 0.03, 0.03, 0.02, 0.14, and 0.12 mmol/L, respectively. In American Indians, rs560887/*G6PC2* had a statistically significant I^2^ p-value for heterogeneity in the association with glucose in American Indians (χ^2^ = 7.31, p = 0.03). For this association, the data came from 3 sites of SHCS (Arizona, Oklahoma, and South Dakota); and the betas were 0.42, -0.01, and 0.17 mmol/L. For each of these SNPs, results of random effects models are presented in Table 3.

## Discussion

Overall, our results demonstrate that all 9 GWAS findings for glucose replicate in EA, yet fewer generalize to other racial/ethnic groups (3/9 in AA, 3/4 in Hispanics, and 2/4 in American Indians and 1/2 in East Asians). In the analysis of insulin, we found that rs780094/*GCKR* replicated in EA, and generalized to Hispanics and American Indians. We observed limited evidence for excess heterogeneity by site in the meta-analyses, with significant heterogeneity detected for only two SNPs.

Some earlier studies have examined the generalizability of some or all of these index SNPs for glucose and insulin in populations of African ancestry, with limited success. The Howard University Family Study [23] found nominal significance (p<0.05) at the *SLC30A8* locus, the Multi-Ethnic Study of Atherosclerosis [24] at *MTNR1B*, and the Candidate Gene Association Resource at *G6PC2, GCK, and MTNR1B*[25]. The sparse generalization of glucose and insulin-related GWAS findings to African Americans could be attributable to several phenomena. First, reduced power in AA: as illustrated in Table 3, several of the non-significant effect sizes in AA were very similar in magnitude to the effect sizes reported in the original GWAS. For example, for rs780094/*GCKR*, the coded allele was associated with similarly higher glucose concentrations in the original GWAS in EA (0.029 mmol/L) [12]. However, the coded allele frequency in PAGE EA was 0.59, versus 0.81 in AA. This difference in coded allele frequency may have reduced our ability to detect an association in AA; the power to detect the previously reported effect size for this SNP was 0.99 in EA, and 0.51 in AA. Second, reduced linkage disequilibrium in AA: lack of association, particularly for analyses having close to adequate power (such as rs560887/*G6PC2* in AA), may be due to differences in linkage disequilibrium patterns between EA and AA. SNPs discovered in GWAS of European populations are largely tagSNPs for causal variants, thus associations between tagSNPs and phenotypes only persist as long as the SNP tagging pattern is upheld. African populations are known to have, on average, much less linkage disequilibrium across the genome than European populations, thus the relationship between European tagSNPs and causal variants may not exist in African populations.

Similar to the PAGE consortium, previous studies have reported nominally significant associations for fasting glucose at the *MTNR1B*, *G6PC2*, and *GCK* loci in Hispanic Americans [24,26], and at *GCKR* in East Asians [27]. Larger follow-up studies are needed to determine whether other genetic regions identified in European GWAS are important in non-Europeans, and to expand research into other populations, such as American Indians, that have not been as thoroughly investigated at these genes.

The PAGE consortium offers a unique opportunity to investigate associations between candidate SNPs and glucose and insulin concentrations in ancestrally diverse cohorts with well-characterized phenotypes. The substantial strength of PAGE is the relatively large samples of ancestrally diverse participants, in which very little is known about the genetic etiology of insulin and glucose concentrations. However, this analysis had several limitations. First, smaller sample sizes in Hispanics (N=3140), American Indian (N=1779), and East Asians (N = 811) limited our ability to detect statistically significant associations in these groups. As shown in Table 2, coded allele frequencies varied considerably between racial/ethnic groups, which likely resulted in reduced power in several analyses. Further, not all SNPs were genotyped in all race/ethnicity groups, which limited our ability to assess generalization of GWAS findings to Hispanics, American Indians, and East Asians. In addition, our genotyping approach was limited to SNPs previously associated with glucose and/or insulin in GWAS conducted prior to 2010. These GWAS were all conducted in European-descent populations, and thus the most promising SNPs from those studies may not be relevant to other populations, due to between-population differences in linkage disequilibrium, particularly in AA.

## Conclusion

In conclusion, in this large and diverse study we were able to replicate 9 GWAS-identified glucose SNPs and 1 of 2 insulin SNPs in EA. Even with limited sample sizes for additional racial/ethnic groups, we found that most of the nine GWAS glucose findings analyzed generalized to at least one non-EA racial/ethnic group, with several SNPs generalizing to multiple groups (e.g., rs780094/GCKR generalized to Hispanics, American Indians, and East Asians) These findings indicate that it would be worthwhile to pursue additional genotype data on larger samples drawn from of these populations, and perform an even more comprehensive investigation of the generalizability of GWAS findings for glucose and insulin in diverse populations. In addition, an investigation of gene-environment and gene-gene interactions may help resolve ancestry-based differences in the genetic basis of glucose and insulin concentrations.

## Competing interests

The authors declare that they have no competing interests.

## Authors’ contributions

MF coordinated the study and drafted the manuscript. JP helped draft the manuscript. PB and SV performed statistical analysis. SB performed quality control procedures on the genotype data. All authors read and approved the final manuscript.

## Pre-publication history

The pre-publication history for this paper can be accessed here:

http://www.biomedcentral.com/1471-2350/14/98/prepub

## Supplementary Material

Additional file 1 Supplementary Methods. Click here for file
