# Supplementary material for: Genetic variants associated with fasting glucose and insulin concentrations in an ethnically diverse population: results from the Population Architecture using Genomics and Epidemiology (PAGE) study
Source: BMC Med Genet. 2013 Sep 25;14:98. doi: 10.1186/1471-2350-14-98 (PMC3849560; doi:10.1186/1471-2350-14-98)
Supplement: Additional file 1 — Supplementary Methods. [file 1471-2350-14-98-S1.doc]

**Title: Genetic variants associated with fasting glucose and insulin concentrations in an ethnically diverse population: results from the Population Architecture using Genomics and Epidemiology (PAGE) Study**

**Supplementary Methods**

*PAGE Cohort Descriptions*

Causal Variants Across the Life Course (CALiCo): CALiCo is a consortium of well characterized population based studies and a central genotyping and resequencing core laboratory. This collaborative network includes six of the most informative and demographically diverse population-based studies extant, contributing approximately 58,000 men and women from the main ethnic and racial groups in the U.S., ranging in age from childhood to old adulthood. Three CALiCo studies are involved in this analysis: Atherosclerosis Risk in Communities Study (ARIC), Cardiovascular Health Study (CHS), and Strong Heart Cohort Study (SHCS).

ARIC is a multi-center prospective investigation of atherosclerotic disease in a predominantly bi-racial population conducted in four U.S. communities, involving both cohort and community surveillance components[1]. European American and African American men and women aged 45-64 years at baseline were recruited from 4 communities: Forsyth County, North Carolina; Jackson, Mississippi; suburban areas of Minneapolis, Minnesota; and Washington County, Maryland. A total of 15,792 individuals participated in the baseline examination in 1987-1989, with follow-up examinations in approximate 3-year intervals, during 1990-1992, 1993-1995, and 1996-1998. Weight and height were measured. Current smoking was dichotomized as yes/no according to the question “Do you now smoke cigarettes?”

The CHS is a prospective, population-based cohort study of cardiovascular disease in older adults[3]. In 1989/1990, a group of 5201 men and women 65 years of age and older was recruited from a random sample of Medicare-eligible residents in 4 US communities: Forsyth County, NC; Sacramento County, CA; Washington County, MD; and Allegheny County, PA. To be considered eligible, persons had to meet the following criteria: 1) age at least 65 years; 2) not institutionalized; 3) expected to remain in the current community for 3 years or longer; 4) not under active treatment for cancer; and 5) provided informed consent without requiring a proxy respondent. To increase the number of African American participants, a supplemental cohort of 687 predominantly African American men and women was recruited in 1992/1993 from three of the same communities (excepting Washington County) using the same sampling and recruitment methods. CHS participants completed standardized clinical examinations and questionnaires at study baselines and at up to 9 annual follow-up visits.

The SHCS is a community-based study of CVD and its risk factors among American Indians, supported by the National Heart, Lung, and Blood Institute (NHLBI) [4, 5]. Research is conducted in three centers (Arizona, South Dakota, Oklahoma). At all centers, some individuals are descended from more than one tribe and/or from non-Indian ancestors.

The Women’s Health Initiative (WHI) is a prospective cohort study investigating post-menopausal women’s health in the U.S [6]. WHI was funded by the National Institutes of Health and the National Heart, Lung, and Blood Institute to study strategies to prevent heart disease, breast cancer, colon cancer, and osteoporotic fractures in women aged 50-79. WHI consists of two parts: randomized clinical trials of hormone therapy, dietary modification, and calcium/Vitamin D supplementation, and an observational cohort study. Subjects for this analysis are 21,000 women drawn from the pool of over 161,000 women involved in either the clinical trial or observational study components of WHI. The 21,000 women were chosen using several selection criteria, which resulted in oversampling women with a BMI > 40, and women belonging to non-white ancestry groups. All selected women were required to have DNA available for analysis.

The Multiethnic Cohort (MEC) is a population-based prospective cohort study of over 215,000 men and women in Hawaii and California aged 45-75 at baseline (1993-1996) and primarily of five ancestries[7]. The MEC was funded by the National Cancer Institute in 1993 to examine lifestyle risk factors and genetic susceptibility to cancer. Cohort members who develop cancer are identified via the California and Hawaii state cancer registries, and all eligible cohort members completed baseline and follow-up questionnaires. Participants eligible for the current analysis were controls in nested case-control studies of breast, colorectal, or prostate cancer or for biomarker studies (N=7,216).

Epidemiologic Architecture for Genes Linked to Environment (EAGLE) accesses the genetic component of three National Health and Nutrition Examination Surveys (NHANES; n~15,000): NHANES III (phase 2 collected between 1991 and 1994), NHANES 1999-2000, and NHANES 2001-2002[8-10]. NHANES is a U.S. population-based, cross-sectional survey of Americans ranging in age from infants to the elderly ascertained regardless of health status, conducted by the National Center for Health Statistics at the Centers for Disease Control and Prevention. In general, DNA samples collected for NHANES on participants 12 years of age or older are linked to survey responses related to demographic, health, and lifestyle as well as data from a physical examination and >100 laboratory measures.

*Data collection*

**BMI**: In EAGLE, all CALiCo studies, and WHI, BMI was calculated from height and weight measured at time of study enrollment in a clinic setting. In WHI only, measurements collected 1 or 3 years after enrollment were substituted for 140 participants missing enrollment height and/or weight. In the MEC, self-reported height and weight were used to calculate baseline BMI.

**Smoking status**: Current smokers were identified in the MEC, WHI, and ARIC by asking subjects whether they were current smokers and in CHS by asking subjects whether they had smoked in the past 30 days. In EAGLE, current smoking for NHANES participants was defined by the answer “yes” to the question “do you smoke cigarettes now?” or if cotinine levels were >15ng/ml. In SHS and SHFS, Tobacco exposure was quantified using standardized questionnaires.

**Racial/ethnic group**: In all studies, self-reported Racial/ethnic group was collected via questionnaire at baseline.

*DNA extraction*

In the MEC and WHI, DNA was purified from buffy coat samples. A subset of MEC DNA samples were whole-genome amplified by Molecular Staging Inc. following their standard protocol [11]. For CALiCo, DNA was extracted from blood samples drawn at baseline. For EAGLE, NHANES III DNA samples are crude cell lysates obtained from Epstein-Barr Virus transformed lymphocyte cell lines [12]. DNA samples from NHANES 1999-2000 and 2001-2002 were purified from blood using standard methods.

*Genotyping*

MEC: Genotyping was conducted at the Cancer Research Center of Hawaii (CRCH) and at the University of Southern California (USC). Genotyping at CRCH was performed using the AB OpenArray genotyping while genotyping at USC was performed using the TaqMan platform. To assess inter-study (within PAGE) and inter-lab (within the MEC) concordance, all SNPs were typed on 375 HapMap samples. The concordance between the CRCH and USC labs was 99.9% for the 8 BMI SNPs genotyped in MEC. In addition, 8.8% blinded duplicate samples were included: resulting in >99.3% concordance.

WHI: Genotyping was performed at the Translational Genomics Research Institute (Phoenix, AZ) on Illumina’s BeadXpress Reader using Illumina’s Veracode GoldenGate genotyping assay, following the manufacturer’s recommended protocol (Reference: [www.illumina.com](http://www.illumina.com/)). Image data were imported into Illumina’s GenomeStudio software. Genotype calls were made using a GenCall cutoff of 0.25. Each batch of ~500 samples included 12 pairs of blind duplicate controls for quality control, with a total of 376 pairs. Overall concordance between pairs was 96.3%.

CALiCo: The majority of genotyping for all sites was performed at the CALiCo central laboratory in Houston, TX using the TaqMan platform using appropriate duplicates and controls. Genotypes were called using the Applied Biosystem Autocaller 3.1 software, subject to manual review. A subset of CHS and ARIC genotypes (rs10838738, rs1333026, rs2815752, rs3751812, and rs8050136) was obtained from previous GWAS studies[13] using either the Illumina 370CNV BeadChip system (CHS) or the Affymetrix Genome-Wide Human SNP Array 6.0 (Santa Clara, CA) (ARIC). For all genotyping platforms, SNPs were excluded if the minor allele frequency was less than 1%, or if they deviated from Hardy-Weinberg expectations (p < 5x10^-5^). Participants were excluded if their genotypes exhibited discordance with prior genotyping, or call rate < 95%. Imputation for four SNPs (rs10838738, rs10938397, rs7498665, and rs3751812) in European American ARIC subjects only was performed using BIMBAM v0.99 with reference to HapMap CEU using release 22, build 36 using one round of imputations and the default expectation-maximization warm-ups and runs. Allele frequencies for imputed SNPs were estimated using the averaged frequencies reported by the other PAGE sites.

EAGLE: For NHANES participants, genotyping was performed by the Vanderbilt DNA Resources Core and the laboratory of Dr. Jonathan Haines, both in Nashville, Tennessee. Genotyping was performed using Sequenom’s iPLEX® Gold coupled with MassARRAY MALDI-TOF MS detection (San Diego, CA) and Illumina’s BeadXpress with a custom GoldenGate assay (San Diego, CA). All NHANES experimental DNA samples and blinded duplicates supplied by CDC were genotyped, and SNPs reported here passed CDC quality control measures and are available for secondary analyses through the National Center for Health Statistics (NCHS) CDC.

**References**

1. *The Atherosclerosis Risk in Communities (ARIC) Study: design and objectives. The ARIC investigators.* Am J Epidemiol, 1989. **129**(4): p. 687-702.

2. Hughes, G.H., et al., *Recruitment in the Coronary Artery Disease Risk Development in Young Adults (Cardia) Study.* Control Clin Trials, 1987. **8**(4 Suppl): p. 68S-73S.

3. Fried, L.P., et al., *The Cardiovascular Health Study: design and rationale.* Ann Epidemiol, 1991. **1**(3): p. 263-76.

4. Lee, E.T., et al., *The Strong Heart Study. A study of cardiovascular disease in American Indians: design and methods.* Am J Epidemiol, 1990. **132**(6): p. 1141-55.

5. North, K.E., et al., *Genetic and environmental contributions to cardiovascular disease risk in American Indians: the strong heart family study.* Am J Epidemiol, 2003. **157**(4): p. 303-14.

6. *Design of the Women's Health Initiative clinical trial and observational study. The Women's Health Initiative Study Group.* Control Clin Trials, 1998. **19**(1): p. 61-109.

7. Kolonel, L.N., et al., *A multiethnic cohort in Hawaii and Los Angeles: baseline characteristics.* Am J Epidemiol, 2000. **151**(4): p. 346-57.

8. National Center for Health Statistics, *Plan and Operation of the Third National Health and Nutrition Examination Survey, 1988-94 (1994) Hyattsville, MD: National Center for Health Statistics. (Vital and Health Statistics, Series 1: Programs and Collection Procedures, no. 32) (DHHS publication no. (PHS) 94-1308) (GPO no. 017-022-01260-0)*.

9. Chang, M.H., et al., *Prevalence in the United States of selected candidate gene variants: Third National Health and Nutrition Examination Survey, 1991-1994.* Am J Epidemiol, 2009. **169**(1): p. 54-66.

10. *National Health and Nutrition Examination Survey (NHANES) DNA Samples: Guidelines for Proposals to Use Samples and Cost Schedule, Centers for Disease Control and Prevention.* Fed Regist, 2010. **75**(108): p. 32191-32195.

11. Dean, F.B., et al., *Comprehensive human genome amplification using multiple displacement amplification.* Proc Natl Acad Sci U S A, 2002. **99**(8): p. 5261-6.

12. Steinberg, K.K., et al., *DNA banking in epidemiologic studies.* Epidemiol Rev, 1997. **19**(1): p. 156-62.

13. Psaty, B.M., et al., *Cohorts for Heart and Aging Research in Genomic Epidemiology (CHARGE) Consortium: Design of prospective meta-analyses of genome-wide association studies from 5 cohorts.* Circ Cardiovasc Genet, 2009. **2**(1): p. 73-80.
